# The Energy Absorption Behavior of 3D-Printed Polymeric Octet-Truss Lattice Structures of Varying Strut Length and Radius

**DOI:** 10.3390/polym15030713

**Published:** 2023-01-31

**Authors:** Matthew Bolan, Mackenzie Dean, Alexander Bardelcik

**Affiliations:** School of Engineering, College of Engineering and Physical Sciences, University of Guelph, Guelph, ON N1G 2W1, Canada

**Keywords:** additive manufacturing, stereolithography (SLA) 3D printing, octet-truss unit cell, energy absorption, failure modes, empirical model

## Abstract

We investigate the compressive energy absorption performance of polymeric octet-truss lattice structures that are 3D printed using high-resolution stereolithography. These structures are potential candidates for personal protective equipment, structural, and automotive applications. Two polymeric resins (high-strength/low-ductility and moderate-strength/high-ductility) were used in this work, and a comprehensive uniaxial tensile characterization was conducted to establish an optimal UV curing time. The external octet-truss structure geometry (3″ × 3″ × 3″) was maintained, and four different lattice cell densities (strut length, L) and three different strut radii (R) were printed, UV cured, and compression tested. The compressive stress–strain and energy absorption (EA) behavior were quantified, and the EA at 0.5 strain for the least dense and smallest R structure was 0.02 MJ/m^3^, while the highest density structure with the largest R was 1.80 MJ/m^3^ for Resin 2. The structural failure modes varied drastically based on resin type, and it was shown that EA and deformation behavior were related to L, R, and the structures’ relative density ($\bar{\rho }$). For the ductile resin, an empirical model was developed to predict the EA vs. compressive strain curves based on L and R. This model can be used to design an octet-truss lattice structure based on the EA requirements of an application.

## 1. Introduction

In contrast with conventional foam/honeycomb cellular metamaterials, energy absorbing lattice structures present a compelling alternative in applications demanding low weight and relatively high stiffness [1,2,3,4]. The applications of such materials vary greatly, from packaging, structural, automotive (panels, energy absorbers, armor), acoustic, and personal protective equipment (PPE) such as sporting helmets [3,4,5,6,7,8], but not medical PPE such as face masks/shields [9]. Another important application of 3D-printed cellular structures includes tissue engineering scaffolds within the medical field [10,11,12].

With the widespread implementation of additive manufacturing (AM) technologies, the production of components with complex and optimized geometries is easily attainable. Due to complex internal lattice geometry, subtractive manufacturing is not viable; therefore, the production of lattice structures using AM methods is preferred. For metallic lattice structures, sacrificial polymeric lattice structures can be 3D printed and used to create a traditional mold to be used in a sand casting [13]. Selective laser melting (SLM) is another metallic AM technique that has seen wide application and attracted research attention within the field of lattice structure manufacture [14,15,16]. Metallic lattice structures offer high energy absorption (EA) capacities, but at the cost of high manufacturing complexity, cost, and increased mass, which reduces their specific energy absorption (SEA) performance. As a result, 3D-printed polymeric lattice structures are receiving increased attention for applications where a high ratio of EA to relative density is desired, and this is especially pertinent in PPE applications where high performance and custom fit equipment is sought [17]. Polymeric lattice structures are produced using a variety of AM technologies, with fused deposition modeling (FDM) [18,19] and stereolithography (SLA) [20] being the most common. FDM 3D printing is particularly attractive for hobbyist applications, such as sporting equipment and art projects, due to its ease of use, low cost of both equipment and filament, and reasonable resolution [21,22]. Due to higher resolution, excellent surface finish quality, isotropic mechanical properties, greater material variety and faster print times, SLA 3D printing is generally preferred, and will be the focus of the current work [3,23,24].

In this work, we focus on repeated-unit cell lattice structures, which have energy absorption applications in PPE, automotive crashworthiness, and packaging. These structures are alternatives to existing cellular materials, which include aluminum honeycombs/foams [20,25,26] and conventional expanded polystyrene foams [26,27,28,29,30]. Unlike the stochastic unit cell structure of cellular materials, the regularity of the lattice unit cell allows one to control the overall structure performance and tune/optimize the lattice geometry to create a functionally graded material for individual design applications [8,14,17,23,26]. Several different unit cell geometries exist and have been examined in the literature [8,18,31,32]. This work focuses on the octet-truss unit cell, which is configured in a face-centered cubic (FCC) arrangement [33]. The strength and stiffness of the octet-truss are stretch-dominant and compare favorably to the corresponding properties of metallic foams, due to the high strength-to-weight ratio, relative ease of manufacture, and potential for multi-functional applications [34]. Numerically, Mueller et al. [32] have shown that octet-truss lattice structures exhibit low variation in stress–strain response with respect to loading orientation and possess a relatively constant stress response over large strains, which is ideal for EA applications. Tancogne-Dejean et al. [35] developed advanced finite element (FE) simulations of stainless-steel octet-truss structures and showed that a unit cell configuration with a relative density of 0.3 (and higher) resulted in a constant (plateaued) compressive stress–strain response, which is a stable and consistent energy absorption profile. They also showed that octet-truss structures with relative densities less than 0.3 resulted in oscillating compressive stress–strain behavior, due to first buckling of the slender struts and followed by a “twist-mode” at the strut nodes for progressive layers of unit cells. Mohsenizadeh et al. [20] examined two different SLA 3D-printed octet-truss lattice structure geometries, whereby the strut diameter was constant, but the strut length was varied to produce structures with relative densities of 0.06 and 0.12. Two different materials (high-strength brittle and low-strength ductile) were also used, and it was shown that the compressive strength and EA increased for the higher relative density and higher strength materials. Micro-cracks resulting from the 3D printing process may have reduced their overall performance. Ling et al. [28] conducted a similar study to [20], but focused on an octet-truss unit cell geometry with a constant strut length, but varied the strut diameter to achieve relative densities of 0.13, 0.26, and 0.41. With respect to the effects of material and relative density on strength and EA, the findings were similar to those in [20]. Additionally, the transition from oscillating to plateaued compressive stress–strain behavior was shown to occur at a similar relative density (0.3) to that shown in [35]. Bardelcik et al. [36] conducted an extensive numerical FE study on the effect that base material properties (due to wash treatment) have on the compressive strength and EA of an octet-truss lattice structure with a relative density of 0.14. They were able to show that tuning the polymeric material properties (post printing) affected the compressive EA profile, leading to a potential to optimize octet-truss performance.

In this work, we utilize high-resolution SLA 3D printing to manufacture several octet-truss lattice structures, for which we vary the unit cell geometry (strut diameter and strut length). Two different polymeric resins were used to print the lattice structures, one that possesses high strength and low ductility, and a second which has moderate strength and high ductility. The printed structures were then compression tested to evaluate the effect of unit cell geometry and base material on the compressive EA performance. We also developed a design tool with which a regression analysis was conducted using the experimental results (ductile resin) to develop an empirical model that can predict EA as a function of octet-truss strut length and radii.

## 2. Materials and Methods

### 2.1. 3D Printing Parameters

**Figure 1:**
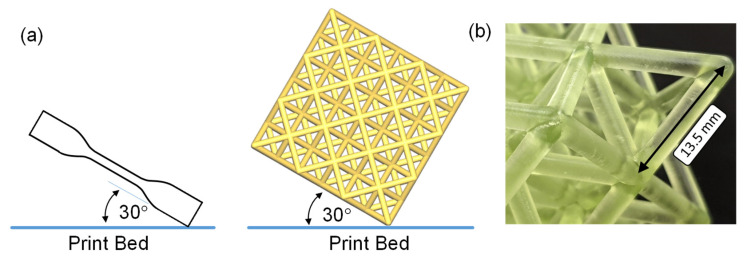
(**a**) A schematic of the dogbone and lattice structure print orientation. (**b**) An image of the high-resolution SLA 3D print quality (Resin 2).

A Phrozen Sonic Mighty 4K SLA 3D printer (Phrozen, Hsinchu City, Taiwan) was used to produce all the specimens in this work. The octet-truss lattice structures consisted of open-faced unit cells ranging in quantity from 27 to 216 (more details in Section 2.4). We also 3D printed uniaxial dogbone specimens for this work. It was found that rotating the dogbone specimen and lattice structure 30° with respect to the print bed resulted in an exceptionally smooth and defect-free surface finish (Figure 1a). In both cases, minimally invasive supports were used during printing and then cut away prior to testing. All the samples were drawn in SolidWorks (Version 2021, Dassault Systemes, Vélizy-Villacoublay, France) and exported as STL files to Chitubox, the Phrozen slicing software (Version 1.9.1, Phrozen, Hsinchu, Taiwan). A layer height of 50 µm was consistently used, and the printer xy resolution was 52 μm. The exposure time for each layer was 3 s.

Following printing, the samples were washed in an ethanol () bath and then cured under UV lighting. Both post-treatments were performed using the Elegoo Mercury Plus (Elegoo, Shenzhen, China). The effect of wash and curing time was evaluated on the dogbone specimens. It was found that wash time had no significant effect on either of the two resins used in this work; therefore, a 5 min wash was used for all of the specimens. The effect of curing time was evaluated on the dogbone specimens for both resins at 0, 5, 15, and 30 min, for which the tensile stress–strain results will be presented in Section 3.1. The results of this study confirmed that a 30 min cure time was optimal for both resins; therefore, all of the 3D-printed lattice structures were washed for 5 min and cured for 30 min prior to testing. All specimens were stored in a dark space to prevent additional curing from ambient light.

### 2.2. Resin Properties

Two different acrylate resins (manufactured by Phrozen, Hsinchu City, Taiwan) were used in this work, the Aqua Blue (referred to as Resin 1) and the Nylon-Green Tough (Resin 2). Resin 1 is a standard hobbyist resin with a relatively high tensile strength and moderate/low total elongation. The composition of Resin 1 consists of urethane acrylated oligomers (65%), acryloyl morpholine (15%), ethoxylated trimethylolpropane tracrylate (15%), diphenyl (2,4,5-trimethylbenzoyl)-phosphine oxide (3%), silica (2%), a photo initiator, and pigment. Resin 2 is classified as an engineering resin with moderate strength, high ductility and consists of a less descript combination of acrylated monomers (0–50%), acrylate-oligomers (10–50%), a photo initiator, and pigment.

### 2.3. Uniaxial Tensile Testing Method

**Figure 2:**
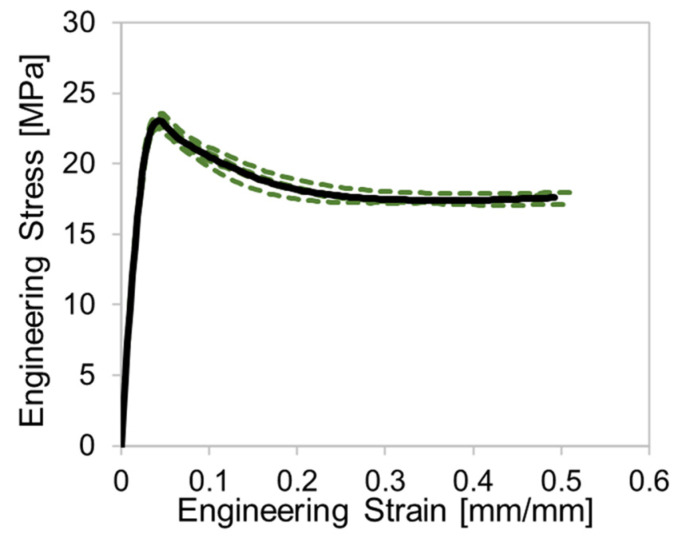
The stress–strain curves for the calculated average (black curve) and individual repeat tests (dashed green curves) for Resin 2 with a 30 min cure time.

To establish the effective of curing time on the materials, tensile tests were performed on the 3D-printed dogbone specimens. The thickness and length of the gauge section was intended to replicate the diameter of the struts in the octet-truss lattices. The dogbone specimens (ASTM D638 Type IV) have a gauge length of 25 mm and a thickness of 2 mm. They were tested in a tensile frame using a clip-on extensometer with a maximum strain measurement up to ~0.50 strain (12.5 mm elongation). It will be shown that Resin 2 exhibited an elongation significantly beyond the range of the extensometer; therefore, we also present elongation data for this material using crosshead displacement as well. The nominal strain rate for all the tests was 0.003 s^−1^. Each cure condition had four repeat tensile tests conducted. The repeatability of the tests was excellent, as shown in Figure 2 (least repeatable case shown) and a single average stress–strain curve was determined via interpolation, as shown by the black curve.

### 2.4. Octet-Truss Geometry and Design

**Figure 3:**
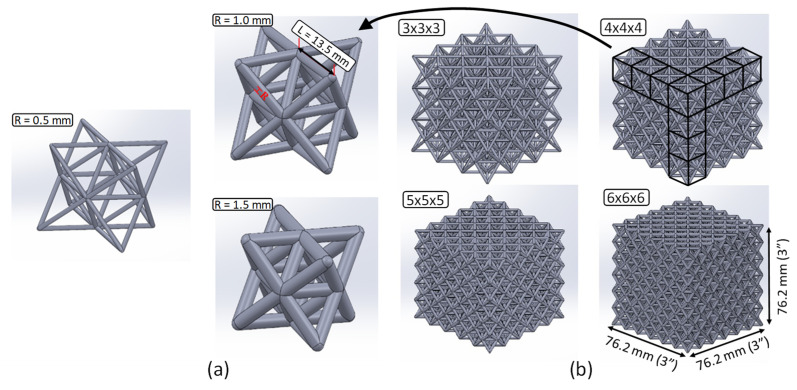
(**a**) Octet-truss unit cell showing the three different strut radii; (**b**) octet-truss lattices in all four cell sizes with a 1.0 mm strut radius.

The octet-truss unit cell consists of an octahedral structure centered in an array of tetrahedral structures positioned along each face of the octet (Figure 3a). The unit cell configuration is that of a face-centered cubic (FCC) arrangement, with the apex of each tetrahedral structure acting as a corner and the vertices of the octet acting as the faces. Stacked in a periodic arrangement, the octet-truss cells form a lattice structure. The size of the cells can be varied along with the exterior dimensions of the lattice to create a variety of unit cell densities (Figure 3b).

For the purposes of this study, the strut length (L) of a given unit cell size is defined as the center-to-center length between the apex of a tetrahedron and any one of the nearest vertices of the octet as shown by the strut length (L) of 13.5 mm in Figure 3a. The strut radius (R) is simply the radius of each strut as shown in Figure 3a and varies from 0.5, 1.0 and 1.5 mm. The exterior dimensions of the cubic lattice structures were consistent throughout the study with a nominal edge dimension of 76.2 mm (3.00 in), as shown in Figure 3b. We chose to consider a range of repeated unit cell densities to fit the constant exterior structure dimension and Figure 3b shows the structures denoted as 3 × 3 × 3, 4 × 4 × 4, 5 × 5 × 5, and 6 × 6 × 6. The 3 × 3 × 3 and 6 × 6 × 6 lattices were produced with three strut radii (R = 0.5, 1.0, 1.5 mm), whereas the 4 × 4 × 4 and 5 × 5 × 5 lattices were produced with the 1.0 mm strut radius as presented in Table 1. Therefore, eight different variants of the lattice structures were printed for each of the two resins. The theoretical relative density (${\bar{\rho }}_{t}$) for each unit cell variation was calculated based on the first-order approximation described in [34]. The average (based on repeat specimens) measured relative density (${\bar{\rho }}_{m}$) was calculated by weighing the printed structures and measuring the overall cubic dimensions, as presented in Table 1 along with ${\bar{\rho }}_{t}$. The published density of Resin 1 and Resin 2 are 1.12 and 1.08 $g/{\mathrm{cm}}^{3}$, respectively.

### 2.5. Octet-Truss Compression Testing Methods

Quasi-static compression tests were performed on the lattice structures using a compression frame. Samples were compressed between two plates at a crosshead velocity of 0.762 mm/s, or a nominal strain rate of 0.01 s^−1^. The samples were compressed until the loading rate increased significantly at the onset of densification.

**Figure 4:**
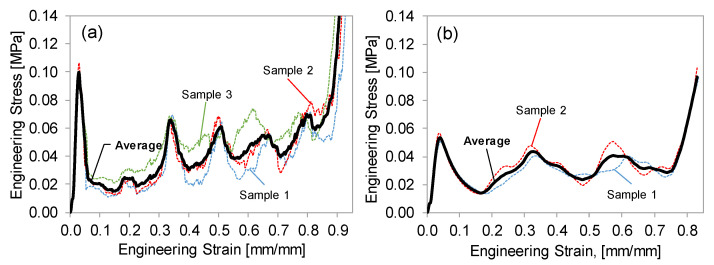
Stress–strain curves for all the individual (dashed) 3 × 3 × 3 R0.5 lattice tests. The bold curve represents the average response (**a**) Resin 1, three samples. (**b**) Resin 2, two samples.

The crosshead extension and compressive load data were converted into engineering (or nominal) stress–strain. The number of repeat tests conducted for each condition and resin are shown in Table 1. As will be discussed in Section 3.2, the high-strength and low-ductility Resin 1 specimens resulted in a less repeatable compression response (resulting in more repeat tests), while the ductile Resin 2 lattice structures exhibited a repeatable response, as shown by the individual test results (dashed curves) in Figure 4. To determine the average response from the population of repeat tests, an interpolation script was used to regularize the strain increment for each repeat test and simply calculate the average stress–strain curve as shown by the bold curve in Figure 4. From this point forward, only the average curves will be presented and used in the analysis.

Utilizing the average nominal compressive stress–strain curves, we determine the total EA by computing the integral of the nominal compressive stress–strain curve,

(1) $$W=\int_{0}^{\varepsilon_{d}}\sigma d\varepsilon$$

where W is the total energy absorbed, *σ* is the average nominal stress, *ε* is the average nominal compressive strain, and *ε_d_* is the nominal compressive strain at which densification of the lattice structure occurs. Densification was taken at the point when the first derivative of stress with respect to strain exceeded the highest such value in the plateau region, and when the second derivative is positive. Furthermore, a qualitative condition was added: the region in which densification can be identified only encompasses the region following the stress peak generated by the collapse of the final lattice row. Mathematically, this can be expressed as:

(2) $$\varepsilon_{d}=\varepsilon \;\mathrm{when}\;\{\begin{array}{c} \frac{d\sigma }{d\varepsilon }>\frac{d\sigma }{d\varepsilon_{pl}}\\ \frac{d^{2}\sigma }{d\varepsilon^{2}}>0 \end{array},\;0.2<\varepsilon_{pl}<0.5,\;\varepsilon_{fp}<\varepsilon$$

where $\varepsilon_{pl}$ is the compressive strain in the plateau region and $\varepsilon_{fp}$ is the compressive strain at the final peak, representing the peak stress of the final lattice row. Contrary to some existing densification criteria, the plateau region was considered to be from 0.2 to 0.5 strain, rather than 0.4 [14,37].

## 3. Results

### 3.1. Uniaxial Tensile Test Results

**Figure 5:**
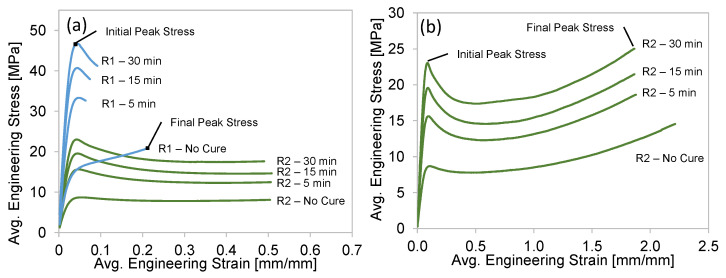
(**a**) The effect of UV cure time on the average stress–strain response for Resin 1 (R1) and Resin (R2). The R2 curves terminate at the maximum range of the clip-on extensometer. (**b**) The average stress–strain response of R2, where the crosshead displacement was used to determine failure strain.

The average tensile engineering stress–strain curves for both resins are shown in Figure 5. Please note that the Resin 2 specimens did not fail at the maximum strain range of the extensometer (~0.5), as shown in Figure 5a. Therefore, the tests were continued beyond this strain level and ultimately to failure, as shown in Figure 5b, where the strain was determined using the crosshead displacement, rather than the extensometer displacement. We recognize that the strains shown in Figure 5b are slightly exaggerated due to the compliance of the tensile frame and deformation outside the specimen gauge length section, but the relatively low strength and small cross-sectional area of the dogbone specimens justifies the presentation of this data.

Considering Resin 1, the uncured material behavior strain hardens continuously beyond a yield strength of ~12 MPa, with a final peak stress of 21 MPa at a total elongation of 0.21. Upon UV curing, significant strengthening was observed with the material exhibiting the expected initial peak stress just after the yield point, followed by a reduction in strength and early fracture. The average initial peak stress/total elongation for the 5, 15, and 30 min cure times were 33/0.06, 41/0.07, and 46 MPa/0.09 strain, respectively. The 30 min cure represented a 124% increase in strength, with a 57% decrease in total elongation. Since the 30 min cure specimen exhibited the highest strength and lowest reduction in ductility (compared to 5 and 15 min), we chose to apply this cure condition to the octet-truss lattice structure for Resin 1.

The uncured and cured Resin 2 material behavior resulted in an initial peak stress that occurred just after yielding, followed by a reduction in strength which eventually increased to a higher final peak stress at the point of total elongation as shown in Figure 5a,b. The average initial/final peak stress of the no cure, 5, 15, and 30 min cure time was 9/15, 16/19, 20/21, and 23/25 MPa, respectively. The total elongation strain at failure was 2.21 for the uncured condition, which decreased to ~1.90 strain for the three different cure times, representing a small 16% reduction. Considering the 67% increase in final peak stress (compared to no cure) and the slight reduction in total elongation, the 30 min cure condition was applied to the octet-truss lattice structure for Resin 2.

### 3.2. Octet-Truss Compression Test Results

#### 3.2.1. Compressive Stress–Strain

**Figure 6:**
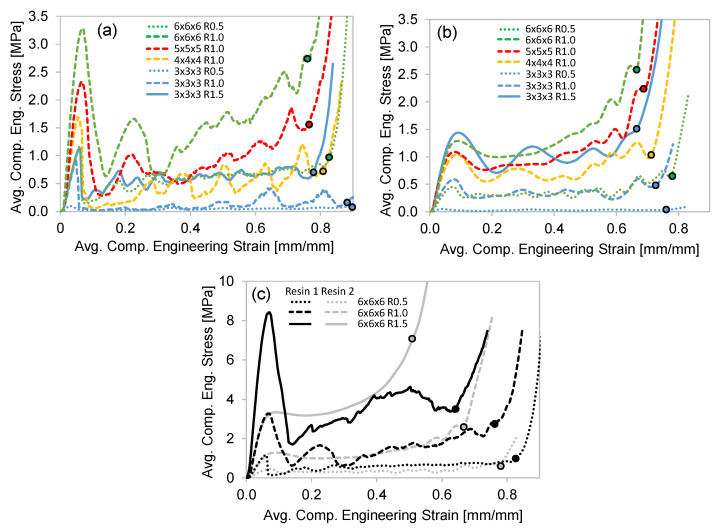
The average compression stress–strain curves for all the lattice samples: (**a**) The results for Resin 1; (**b**) the results for Resin 2; (**c**) a comparison of the Resin 1 and 2 results for the 6 × 6 × 6 lattice structures. The circular data point indicates the onset of densification.

The average compression stress–strain curves for all lattices and both resins are shown in Figure 6. The onset of densification is determined using the Equation (2) criteria and shown by the circular data points.

**Figure 7:**
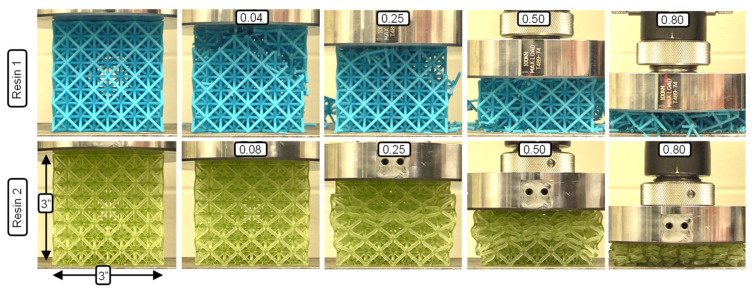
Compression test images for the 4 × 4 × 4 R1.0 lattice structures.

The Resin 1 results exhibit a strong peak stress in the range of 0.04–0.07 strain for all the lattice structures tested (Figure 6a,c), followed by a sharp reduction in stress. This behavior is due to the initial elastic loading of the entire structure, which is followed by the sudden failure of the low ductility struts. Beyond the peak stress, further compression results in an oscillating stress behavior which can be seen for the individual test in Figure 4a. The oscillations (stress rise and drop) occur as successive layers of the unit cells fail for this 3 × 3 × 3 R0.5 case, which represents the lowest relative density. The jagged stress response of the repeat tests shown in Figure 4a are a result of individual strut failures. For higher unit cell densities, initial failure was sporadic and at times occurred through multiple layers, as shown in Figure 7, just after peak stress. This behavior resulted in an unrepeatable stress–strain response between repeat tests, making the compressive properties of these lattice structures undesirable, especially for structures with higher relative densities. There is a clear strengthening in stress–strain response for increased strut radius (R) with respect to a constant unit-cell density as shown for the 3 × 3 × 3 (Figure 6a) and 6 × 6 × 6 (Figure 6c) lattice structures. The strengthening effect of increased unit-cell density, or decreased strut length (L) for a given strut radius is evident for the R1.0 structures shown in Figure 6a.

With respect to the effect of L and R, the Resin 2 compressive stress–strain behavior is aligned with that of Resin 1, but with some notable differences. The two major differences are that (1) none of the Resin 2 structures exhibited strut failure, and (2) a reduced initial peak stress was observed for the Resin 2 structures. The peak stress is relatively low (compared to Resin 1) and signifies the elastic response of the overall lattice structure (see Figure 7, 0.08 strain) just prior to individual layers of unit cell collapse. It should be noted that we observed the same twist mode of unit-cell collapse for structures with relative densities less than ~0.3, and a stable mode of collapse for lattice structures with higher relative densities as discussed in [35]. This results in a repeatable and oscillating stress response for the twist-mode collapse (see Figure 4b) which occurs layer-by-layer and a smooth (or plateaued) stress response for the stable deformation as shown for the 6 × 6 × 6 R1.5 and R1.0 structures in Figure 6c. Similar strengthening trends with respect to increases in unit-cell density (reduced L) and strut radius were observed. It must also be noted that due to the absence of strut failure, the Resin 2 structures recovered a percentage of their original height (not quantified) after being loaded beyond the point of densification, as discussed in [20]. This contrasts with the Resin 1 structures, which fail catastrophically during deformation and did not recover at all. This result is important for the potential applications of these structures in PPE designs. If the energy absorbing application considers a single impact, the Resin 1 structures may be appropriate.

#### 3.2.2. Compressive Energy Absorption

**Figure 8:**
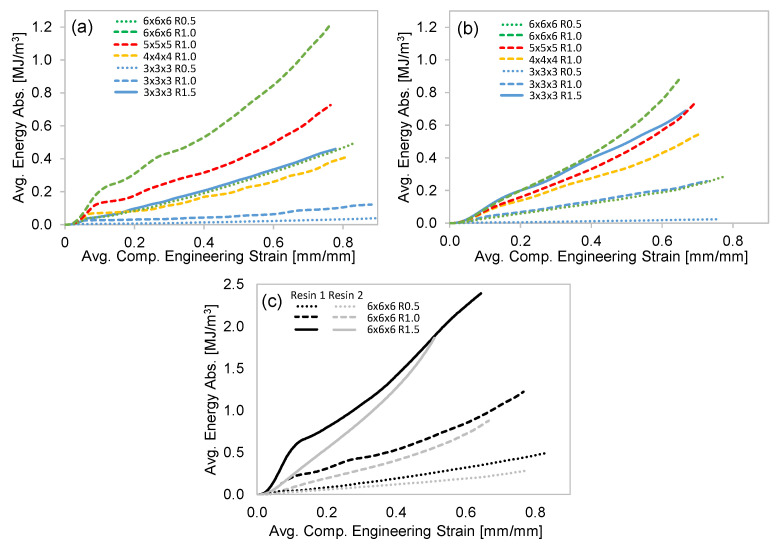
The average compression EA curves for all the lattice samples: (**a**) the results for Resin 1; (**b**) the results for Resin 2; (**c**) a comparison of the Resin 1 and 2 results for the 6 × 6 × 6 lattice structures. All curves terminate at the point of densification.

The average EA curves for all the lattice structures are shown in Figure 8. The data presented in this figure are normalized by the specimen volume (resulting in energy density), and the curves terminate at the onset of densification, as shown by the data points in Figure 6. All the curves monotonically increase, but the initial rate of the Resin 1 curves is steep due to the higher peak stress, which is less evident for the Resin 2 curves. As expected, the level of EA is greater for lattice structures with a stronger stress–strain response and the same trends with respect to increased strut radius (R) and reduced strut length (L) resulted in greater EA for a given strain level. When comparing the EA for the 6 × 6 × 6 structures (Figure 8c), the Resin 1 curves show more EA and a greater strain at which densification occurs. A major contribution to the increased EA levels in Resin 1 is the initial rate of EA, which is a result of the high initial peak stress of the material.

## 4. Discussion

### 4.1. Uniaxial Tensile Material Properties

**Figure 9:**
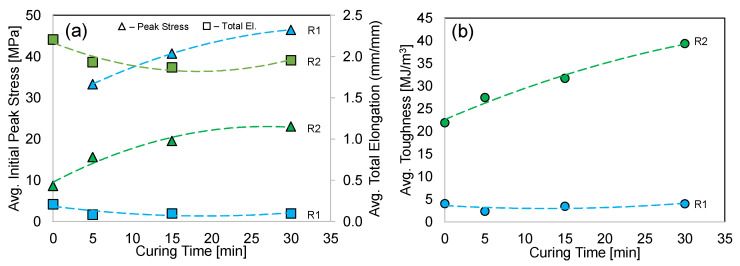
The effect of curing time on the uniaxial tensile performance of Resin 1 (R1) and Resin 2 (R2): (**a**) average initial peak stress and total elongation strain; (**b**) average toughness, computed from the tensile stress–strain curves. Regression analysis was used to fit polynomial trendlines to the data.

The uniaxial stress–strain curves for the two resins are shown in Figure 5, and a summary of the effect that curing time has on the initial peak stress, total elongation and toughness is shown in Figure 9. The initial peak stress of Resin 1 is approximately double that of Resin 2 for all curing times. This is an excellent result if we consider this peak stress being synonymous with the yielding point of these polymers. For applications where the expected load is below the point of yielding, then Resin 1 is the superior choice. This is clearly shown in the compression test results of the Resin 1 lattice structures in Figure 6a, where the initial peak stress just prior to failure of the structure struts is high and aligns with the data in Figure 9a. There appears to be an opportunity to further strengthen Resin 1 (increasing trendline projection) with additional curing beyond the 30 min examined in this work, whereas the Resin 2 initial peak stress appears to be nearing a saturation point beyond the 30 min mark. 

Although the initial peak stress is high for Resin 1, the tensile test total elongation strain is very low when compared to Resin 2. As a result, the limited ductility of the Resin 1 tensile specimens resulted in a low EA potential, which is quantified as the toughness (or area under stress–strain curve) in Figure 9b. As the initial peak stress of this material increased, the total elongation strain decreased, resulting in toughness value of ~3 MJ/m^3^, for all the curing times. In stark contrast to this result, the Resin 2 total elongation strain was nearly unchanged for the cured specimen tests, therefore, combined with the increased initial peak stress (and high final peak stress) for higher curing times, the toughness of the Resin 2 material increases for greater curing times as shown in Figure 9b. The average toughness for a 30 min cure is 39 MJ/m^3^, and the trendline fit to the data suggests that the toughness can be further enhanced with longer curing.

### 4.2. Octet-Truss Compression Tests

#### 4.2.1. Deformation Modes and Energy Absorption

**Figure 10:**
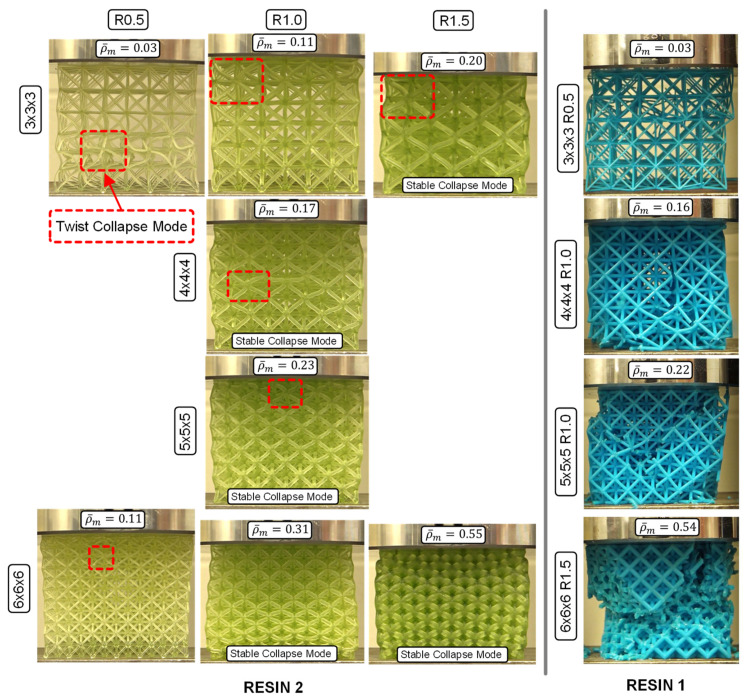
The failure modes observed for the Resin 1 and 2 octet-truss structures.

Figure 10 shows the deformation modes for all the Resin 2 lattice structures tested in this work. Based on the description of unit cell deformation (or collapse) modes in [35], a pure twist-mode collapse was observed for the three structures with the lowest measured relative density (${\bar{\rho }}_{m}$) (0.03, 0.11, 0.11), where an entire layer of unit cells collapsed, compressed fully, and then engaged the next layer of unit cells. This resulted in the oscillating stress–strain behavior shown in Figure 4b and Figure 6b for these low ${\bar{\rho }}_{m}$ structures. The twist mode of collapse is a result of the slender struts buckling and forming a hinge at their center, as shown in Figure 10. The two lattice structures with the highest relative density (0.31 and 0.55) collapsed in a stable deformation mode, where the width of the structures expanded laterally during deformation. This stable deformation mode was due to the higher strut radius-to-length ratio, which created hinge points at the two strut nodes (rather than at the center), resulting in a smooth stress–strain response, as shown in Figure 6c. The remainder of the lattice structures (${\bar{\rho }}_{m}$ = 0.17, 0.20, 0.23) exhibited characteristics of a combined twist and stable collapse mode. Again, these results are in agreement with the predicted octet-truss lattice structure behavior presented in [35], where the transition between twist and stable collapse deformation was found to occur at a relative density of ~0.3.

Due to the catastrophic failure behavior of the Resin 1 lattice structures (see Figure 7 and Figure 10), it was difficult to observe the same deformation modes as described for Resin 2. The twist collapse mode was only observed for the lowest ${\bar{\rho }}_{m}$ structure (3 × 3 × 3 R0.5), which exhibited only a slight twist, prior to brittle fracture of the struts due to the low material ductility. As discussed previously, the large stress oscillations were a result of individual layers of unit cells failing, where the smaller oscillations (or jagged line profile) were attributed to individual strut failures as shown in Figure 4a. The remainder of the Resin 1 structures displayed very sporadic failure modes during compression. The lower ${\bar{\rho }}_{m}$ structures typically exhibited a combined failure mode that included initial failure through multiple unit cell layers and a progressive unit cell layer failure mode, as shown for ${\bar{\rho }}_{m}$ = 0.16 and 0.22 in Figure 10. The higher-relative-density 6 × 6 × 6 R1.5 (${\bar{\rho }}_{m}$ = 0.54) structure behaved more sporadically, and resulted in the ejection of entire sections of the octet truss due to a lack of containment during compression. Figure 6c shows the stress–strain curve for the 6x6x6 R1.5 structure, and it is expected that the stress beyond the peak stress would have been greater if containment of the structure had occurred.

**Figure 11:**
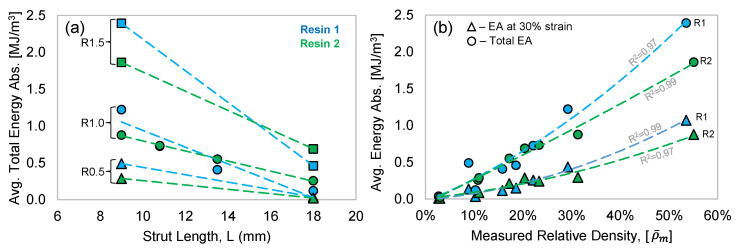
(**a**) The effect or strut length (L) and radius (R) on the average total EA. Dashed lines represent a linear regression fit to the data. (**b**) The average EA with respect to the measured relative density (${\bar{\rho }}_{m}$). Regression analysis was used to fit polynomial trendlines to the data.

In Figure 11a, the effect of L and R on the total EA of the structures tested in this work is quantified. The trends clearly show that the total EA increases with shorter strut lengths, or increased unit cell density. This trend can be observed for each strut radius value, but the relative difference in EA increases with increasing strut radius. For example, in Resin 2, the total EA increase (L = 18 to 9 mm) for R0.5 is 0.02 to 0.29 MJ/m^3^ (+1132%), while the R1.5 total EA increases from 0.69 to 1.86 MJ/m^3^ (+170%). A similar trend exists for the total EA of Resin 1, but the sporadic fracture behavior and the assessment of total EA leads to less confidence in the results. Nonetheless, Figure 11a reveals that the total EA trends with respect to R and L are somewhat similar for both resin materials, even though they represent very different material properties and deformation modes during compression. This can again be attributed to Resin 1 having higher strength and low ductility, which results in elastic high peak stress and stress oscillations, as the struts catastrophically fail during deformation. Resin 2, on the other hand is a moderate-strength material, but due to its exceptional ductility, which prevents strut failure, the struts continuously absorb energy, whether they exhibit the twist or stable collapse deformation mode.

The data presented in Figure 8 and Figure 11a can also be related to ${\bar{\rho }}_{m}$, as shown in Figure 11b. Regression analysis revealed that a polynomial function fit the data very well, as indicated by the R-squared (goodness of fit) values shown in the figure. The EA at a compression strain of 0.3 is plotted for both resins, and shows the expected increase in EA with respect to ${\bar{\rho }}_{m}$. The Resin 1 and Resin 2 data are somewhat identical for ${\bar{\rho }}_{m}$ < 0.3, while for the structures with higher ${\bar{\rho }}_{m}$, the EA of Resin 1 is greater due to the higher initial peak stress (Figure 6c) of the Resin 1 structures, which results in a higher initial EA response, as shown in Figure 8c. The total EA (data from Figure 11a) results show an excellent fit with respect to ${\bar{\rho }}_{m}$ for Resin 2. The Resin 1 total EA is greater than that of Resin 2 for higher ${\bar{\rho }}_{m}$, which is again attributed to the initial peak stress behavior of Resin 1 and the higher densification strain for the Resin 1 structures due to the catastrophic failure and ejection of pieces of the structure. The Resin 1 total EA for ${\bar{\rho }}_{m}$ = 0.09 (6 × 6 × 6 R0.5) is an outlier resulting from a higher-than-expected densification point due to the failure mode of the brittle struts. The EA results presented in Figure 11a,b indicate that the effect of R and L on EA can be related to the structures’ ${\bar{\rho }}_{m}$, which can be calculated using R and L. 

#### 4.2.2. Stress–Strain and Energy Absorption Comparison to EPS Foams

**Figure 12:**
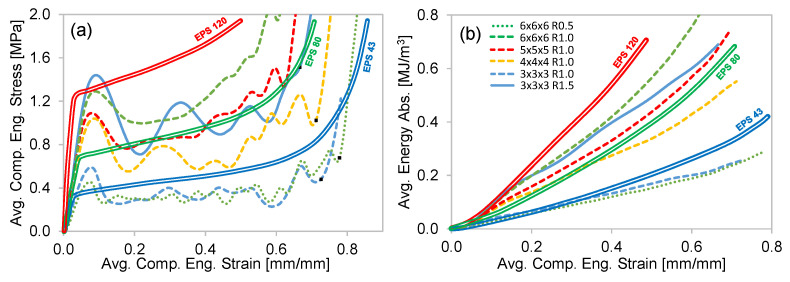
A comparison of the (**a**) stress–strain and (**b**) EA characteristics between Resin 2 octet-trusses and EPS foams. The legend is common to both graphs. The EPS foam values were digitized and computed from [27].

Expanded polystyrene (EPS) foams are commonly used energy absorbing materials in PPE applications. Ling et al. [27] conducted compression tests on a variety of EPS foams that varied in density from 43 to 120 kg/m^3^. The compressive stress–strain behavior of these foams is compared to the Resin 2 octet-truss structures in Figure 12a. The EPS foams do not exhibit an initial peak stress and have a relatively consistent and increasing plateau region up to the point of densification. Depending on the unit cell density and strut radius, very similar stress–strain behavior can be achieved by the octet-truss structures examined in this work. For example, the EPS 43 response (although slightly higher) is like that of a 3 × 3 × 3 R1.0 (${\bar{\rho }}_{m}=0.10$) and 6 × 6 × 6 R0.5 (${\bar{\rho }}_{m}=0.09$) structure. These two octet-truss structures are geometrically very different (see Figure 10), which allows one to select and implement an octet-truss configuration based on geometric design constraints. It should also be noted that the higher-relative-density unit cell structures (6 × 6 × 6 and 5 × 5 × 5) exhibit fewer stress oscillations, replicating the EPS behavior more closely.

The average EA curves for the compression stress–strain tests examined in Figure 12a are shown in Figure 12b. Due to the similarity in stress–strain behavior, the octet-truss EA response is like that of the EPS foams. The initial peak stress of the octet-truss structures results in a somewhat higher initial rate of EA, which then behaves like EPS during steady-state deformation. This is illustrated by the EPS 80 and 5 × 5 × 5 R1.0 curves, where the initial peak stress is considerably higher for the octet-truss up to ~0.10 strain, but then almost identical for the remainder of the test. This initial peak stress results in an offset in EA at ~0.10 strain, which is then maintained as shown by the two curves in Figure 12b, which appear to be offset by a constant value up until the end of the test.

It has been shown that the stress–strain and EA behavior of the Resin 2 octet-truss structures can sufficiently match that of EPS foams. Although this is a good result, the mass of the structures must also be considered for PPE applications. The specific energy absorption (SEA) is calculated by dividing EA by the structure mass and it has been shown the EPS foams have a higher SEA than 3D-printed octet-truss structures due to their low density [28]. This observation was also made for the octet-truss structures examined in the current work. Considering this factor, octet-truss structures are applicable to higher weight PPE and other energy absorption applications, such as in packaging, structural and automotive applications. One of the main advantages of 3D-printed octet-truss structures is the ability to combine different unit cell densities and strut radii within a single 3D-printed structure, which are referred to as functionally graded lattice structures [14]. This allows one to locally tune the EA behavior of a larger structure based on design constraints and optimize the overall mass of the final product. Another opportunity for improved SEA is the potential to 3D print octet-truss structures with hollow struts [38]. Hollow struts (vs. solid) were shown to reduce the EA, but increase the SEA as a result of lightweighting. The best potential method of improving SEA is via resin development and post-printing treatments (e.g., washing [36] and curing) to increase the strength of these materials without sacrificing ductility. Additionally, the octet-truss structures (Resin 2 only) presented in the current work are able to recover their original geometry (see [20], as well), since no strut failure/fracture was observed. This property is advantageous over EPS, which is not able to recover its shape after deformation, resulting in a single-impact use/functionality. 

### 4.3. Empirical Energy Absorption Model 

The Resin 2 EA curves (Figure 8 and Figure 12) are important design datasets when considering these structures for functional engineering applications. As a result, we utilized the experimental data generated in this work to develop a mathematical model to predict the EA vs. compressive strain curves for any strut radius (R) and strut length (L).

**Figure 13:**
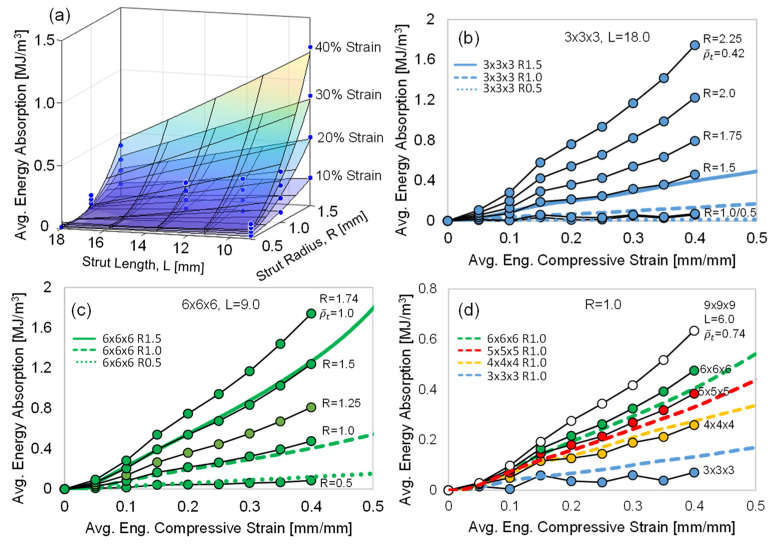
(**a**) A plot of Equation (3) for various compressive strain increments. The circular data points represent the experimental data. The predicted and measured energy absorption curves for (**b**) 3 × 3 × 3, L = 18.0; (**c**) 6 × 6 × 6, L = 9.0; and (**d**) R = 1.0, various L (unit cell density). The circular data points in (**b**–**d**) are predicted, and the continuous curves are the measured data.

Using non-linear regression analysis, a polynomial three-dimensional surface function (Equation (3)) was fit to the experimental average EA [MJ/m^3^] data, where the independent variables were R and L. The regression analysis was conducted at compressive strain increments of 0.05, up to 0.40 strain, resulting in eight separate fits, for which the polynomial constants ($\beta_{1-6}$) in Equation (3) were unique at each strain increment. We elected to use the experimental data up to 0.40 compressive strain due to the early densification of the 6 × 6 × 6 R1.5 structure, as shown in Figure 6c. The resultant surface plot fits of Equation (3) are shown in Figure 13a for 0.10 compressive strain increments.

Each of the Equation (3) constants ($\beta_{1-6}$) was then plotted with respect to compressive strain (0.05 to 0.40 at 0.05 increments), and a non-linear regression analysis was conducted to fit each constant with respect to compressive strain, using a polynomial function, as presented in Table 2 for $\beta_{1-6}\left(\varepsilon \right)$. Some of the constants required a 6th-order polynomial fit ($\beta_{1}\left(\varepsilon \right)$ and $\beta_{3}\left(\varepsilon \right)$), while others were fit with a simple 1st-order linear function. The R^2^ values were excellent for all the fits as shown in Table 2. The compressive strain (ε) can now be incorporated into Equation (3) as a third independent variable, resulting in the final form of the EA model, Equation (4).

Equation (4) can now be used to predict the EA vs. compressive strain curves for any strut radius (R) and length (L) as shown in Figure 13b–d. The 3 × 3 × 3 (L = 18) and 6 × 6 × 6 (L = 9) experimental data were accurately predicted, as shown in Figure 13b,c. For the R = 1.0 results (Figure 13d), the predicted experimental results were good, with a slight underprediction of the 3 × 3 × 3 results and a minor overprediction of the 5 × 5 × 5 and 6 × 6 × 6 results. The reduced predictability of the model in the R = 1.0 case is partially due to the relatively low EA for structures with this particular strut radius, as indicated by the low maximum value of the EA axis shown in this graph. Also plotted in Figure 13b,c are predicted EA curves for higher R values. For the 6 × 6 × 6 structure, where the theoretical relative density (${\bar{\rho }}_{t}$) becomes 1.0 for a strut radius of 1.74 mm, there is minimal potential to increase the EA capacity of this high-density octet-truss configuration. Due to the long strut length of the 3 × 3 × 3 structure, the radius can be increased significantly and the predicted EA curve for R = 2.25 suggests that a ~300% (at 0.40 strain) increase is possible and further increases may be viable since ${\bar{\rho }}_{t}$ = 0.42 for this case. The potential increase in EA of the R = 1.0 (Figure 13d) is likely minimal, as the predicted EA of the 9 × 9 × 9 (L = 6.0, ${\bar{\rho }}_{t}$ = 0.74) case is only ~30% greater, with a relative theoretical density approaching 1.0. The authors recognize that the model was developed based on a strut radius and length of 0.5 ≤ R ≤ 1.5 and 9 ≤ L ≤ 18; therefore, predicting the EA outside of these boundary conditions has not been validated at this time and requires further research.

(3) $$f\left(R,L\right)=\beta_{1}+\beta_{2}R+\beta_{3}L+\beta_{4}R^{2}+\beta_{5}RL+\beta_{6}L^{2}$$

(4) $$f\left(R,L,\varepsilon \right)=\beta_{1}\left(\varepsilon \right)+\beta_{2}\left(\varepsilon \right)R+\beta_{3}\left(\varepsilon \right)L+\beta_{4}\left(\varepsilon \right)R^{2}+\beta_{5}\left(\varepsilon \right)RL+\beta_{6}\left(\varepsilon \right)L^{2}$$

## 5. Conclusions

The effects of 3D-printed octet-truss geometry (strut radius and length) and resin material properties were investigated in this work. Based on a comprehensive assessment of the compressive stress–strain, energy absorption and deformation behavior of the lattice structures tested in this work, the following conclusions can be made:

- High-resolution SLA 3D printing was used to produce specimens with high geometric accuracy and no visible defects. This insured that the tensile/compression test results were not influenced by print defects.
- Two different acrylate resin blends were used to print and test uniaxial tensile specimens. After a 30 min UV cure, Resin 1 exhibited a peak strength increase to 46 MPa (+124% compared to uncured) and a decreased total elongation strain to 0.09 (−57% compared to uncured). The post-cure peak strength of Resin 2 increased to 23 MPa (+67%), and the total elongation strain decreased slightly to 1.86 (−16%). There is the potential for further increases in strength with additional curing.
- Compression tests for the Resin 1 octet-truss structures exhibited a strong initial peak stress (maximum of 8.3 MPa for 6 × 6 × 6 R1.5) that was followed by a sudden strength reduction because of the catastrophic strut failure of the low-ductility material. Only the structure with the lowest relative density ($\bar{\rho }$) failed layer-by-layer, while the other structures failed through multiple layers, resulting in sporadic stress–strain behavior. There is a clear increase in the compressive stress–strain behavior with decreasing strut length L and increasing strut radius R, which is also captured by an increase in $\bar{\rho }$.
- Similar increases (due to increased $\bar{\rho }$) in compressive stress–strain behavior were shown for the Resin 2 structures, but the exceptional ductility of this resin resulted in no strut failure, and the severe initial peak stress (shown for Resin 1) was not observed, while a maximum initial stress of 3.3 MPa still resulted for the 6 × 6 × 6 R1.5 structure. The deformation mode transitioned from a twist mode (oscillating stress response), for structures with a low $\bar{\rho }$, to a stable collapse mode (plateaued stress response), at the highest values of $\bar{\rho }$.
- For both resins, the increase in EA was clearly related to reduced L and increased R. It was also shown that, despite the large difference in strength and ductility between the two resins, the total EA was similar with respect to L and R for both resins. For Resin 2, the EA at 0.5 compressive strain for the highest L and smallest R structure (3 × 3 × 3 R0.5) was 0.02 MJ/m^3^, while the structure with the highest L and smallest R (6 × 6 × 6 R1.5) was 1.86 MJ/m^3^. This difference was similar for the Resin 1 structure. When plotted against relative density, the EA data for both resins fit a polynomial trend very well, which is related to R and L.
- The Resin 2 EA versus compressive strain experimental data was used to develop an empirical model that can be used to predict the EA versus compressive strain curves for any R and L, within the bounds of the data that was used to develop the model. This can be used as a design tool for predicting octet-truss EA properties.

## Figures and Tables

**Table 1:** Summary of the lattice structure geometry and relative density.

Lattice Structure Density	Strut Length, L [mm]	Strut Radius, R [mm]	Number of Specimens, Resin 1, Resin 2	Theoretical Relative Density, ${\bar{\rho }}_{t}$	Resin 1 (Blue), Avg. Meas. Rel. Density, ${\bar{\rho }}_{m}$	Resin 2 (Green), Avg. Meas. Rel. Density, ${\bar{\rho }}_{m}$
3 × 3 × 3	18.0	0.5	3, 2	0.02	0.03	0.03
	18.0	1.0	2, 2	0.08	0.10	0.11
	18.0	1.5	1, 2	0.19	0.19	0.20
4 × 4 × 4	13.5	1.0	3, 2	0.15	0.16	0.17
5 × 5 × 5	10.8	1.0	3, 2	0.23	0.22	0.23
6 × 6 × 6	9.0	0.5	1, 2	0.08	0.09	0.11
	9.0	1.0	2, 1	0.33	0.29	0.31
	9.0	1.5	2, 2	0.74	0.54	0.55

**Table 2:** The polynomial constants used in Equation (4).

	$\varepsilon^{0}\;$	$\varepsilon^{1}\;$	$\varepsilon^{2}\;$	$\varepsilon^{3}\;$	$\varepsilon^{4}\;$	$\varepsilon^{5}\;$	$\varepsilon^{6}$	R-Squared Value
$\beta_{1}\left(\varepsilon \right)$	−412.5	18,739.2	327,039.6	2,569,483.8	−10,103,339.0	19,292,288.6	−14,228,271.0	0.99
$\beta_{2}\left(\varepsilon \right)$	−38.4	1121.7	−	−	−	−	−	0.99
$\beta_{3}\left(\varepsilon \right)$	73.5	−3274.8	55,856.4	−430,961.3	1,664,155.3	−3,124,910.8	2,269,996.2	1
$\beta_{4}\left(\varepsilon \right)$	44. 7	−1760.4	40,872.2	−209,484.0	489,014.6	−414,169.6	−	1
$\beta_{5}\left(\varepsilon \right)$	4.7	−150.8	−178.3	−	−	−	−	1
$\beta_{6}\left(\varepsilon \right)$	0.3	−8.3	−4.1	557.7	−2409.5	2970.7	−	0.98

## Data Availability

The data presented in this study are available on request from the corresponding author.

## Abbreviations

**Abbreviation**	**Meaning**
AM	Additive manufacturing
EA	Energy absorption
FCC	Face-centered cubic
FDM	Fused deposition modeling
FE	Finite element
PPE	Personal protective equipment
SEA	Specific energy absorption
SLA	Stereolithography
SLM	Selective laser melting
UV	Ultraviolet
